# Adult‐onset myoclonus ataxia associated with the mitochondrial m.8993T>C “NARP” mutation

**DOI:** 10.1002/mds.26358

**Published:** 2015-08-12

**Authors:** Mika H. Martikainen, Grainne S. Gorman, Paul Goldsmith, David J. Burn, Doug M. Turnbull, Andrew M. Schaefer

**Affiliations:** ^1^ Wellcome Trust Center for Mitochondrial Research, United KingdomNewcastle UniversityNewcastle upon TyneUnited Kingdom; ^2^ Institute of NeuroscienceNewcastle UniversityNewcastle upon TyneUnited Kingdom

Mitochondrial dysfunction is an important cause of neurological disease. Mitochondrial disease can present at any age, and the clinical features are extremely varied. The mitochondrial DNA (mtDNA) m.8993T>C mutation is usually encountered either in association with Leigh syndrome^1^ or the syndrome of neuropathy, ataxia, and retinitis pigmentosa (NARP).^2^ We describe severe, late‐onset myoclonus ataxia related to the m.8993T>C mutation.

A 74‐year‐old man presented with a 3‐year history of progressive hand clumsiness and poor balance. He was now wheelchair‐bound and needed assistance in dressing, feeding, and washing. There was no history of seizures. He had a sister with diabetes mellitus and coeliac disease, but otherwise well. Family history was unremarkable for movement disorders and other neurological conditions. Both the patient and his sister had no children, and their mother who was deceased was an only child.

Clinical examination revealed bilateral, irregular, and multifocal myoclonus in both upper limbs, considerably aggravated by active movement. No pigmentary retinopathy was detected. Up‐gaze was limited, but with intact smooth pursuit. Cognition was normal. Proximal (MRC 4/5) and distal (MRC 4+/5) muscle weakness was evident. Lower‐limb reflexes were absent. Severe myoclonus ataxia rendered him unable to walk or stand independently.

Urea, liver function tests, creatinine kinase, serum lactate, vitamin E, blood copper, and coeruloplasmin levels were unremarkable. Testing for anti‐gliadin and anti‐endomysial antibodies was negative.

Brain MRI revealed global cortical and cerebellar atrophy. Nerve conduction studies revealed a predominantly sensory axonal neuropathy. EEG did not reveal any epileptiform features. Photic stimulation evoked further myoclonic jerks associated with muscle activity artefact.

Fragile X–associated tremor ataxia syndrome and spinocerebellar ataxia types 1 to 3, 6, 7, and 17 were excluded; *SGCE* gene analysis was normal. Given that clinical features were suggestive of a mitochondrial disorder rather than other causes of myoclonus ataxia, common *POLG* mutations (p.Ala467Thr, p.Trp748Ser, and p.Gly848Ser) and mtDNA point mutations associated with MERRF (myoclonic epilepsy with ragged‐red fibers) syndrome (m.8344A>G, m.8356T>C, and m.8363G>A) were excluded. Pyrosequencing of the *MTATP6* and *MTATP8* genes (methodology as described by Alston et al.^3^) revealed the m.8993T>C mutation in *MTATP6* at 95% heteroplasmy in the patient's blood and urine. The m.8993T>C mutation was undetectable in the respective samples from his sister, suggesting a sporadic mutation, although a maternal sample was not available to confirm this.

Myoclonus linked with ataxia is a common feature of the m.8344A>G MERRF mutation,^4^ and it has been reported with *POLG* mutations.^5^ Adult‐onset ataxia and axonal neuropathy owing to the m.8993T>C mutation has been reported in a Finnish family,^2^ but severe myoclonus ataxia in association with the m.8993T>C mutation has not been previously reported. The patient's late symptom onset is remarkable. Clinical clues suggesting a possible late‐onset mitochondrial disorder were the multisystem nature of the disorder and a phenotype characterized by myoclonus ataxia, neuropathy, and the mild up‐gaze limitation. Cerebellar pathology and ataxia are common features of mitochondrial disease. Recently, the cerebellum has been implicated in the pathogenesis of cortical myoclonus.^6^ Apart from mitochondrial disease, myoclonus ataxia can be associated with many other conditions, such as coeliac disease, and various rare metabolic or genetic disorders.^7^ Mitochondrial disease related to the m.8993T>C mutation should be considered among adult patients who present with nondominant myoclonus ataxia, irrespective of age.

## Legends to the Videos

**Video 1.** This video was taken at the age of 73 years. The sensitivity of the myoclonic jerks to photic stimulation is demonstrated during the EEG recording.

**Video 2.** This video was taken at the age of 74 years. The severe myoclonus ataxia is demonstrated during the finger‐to‐finger test and when trying to pick up a pen. On examination of the eye movements, smooth pursuit is intact, but there is evidence of marked upward gaze restriction. The head drop is owing to neck extensor weakness (MRC 4/5) rather than dystonia.


Mika H. Martikainen, MD, PhD,^1^ Grainne S. Gorman, MRCP, FRCP,^1^ Paul Goldsmith, MRCP,^2^ David J. Burn, FRCP,^2^ Doug M. Turnbull, FRCP,^1^ and Andrew M. Schaefer, MRCP^1^
^1^Wellcome Trust Centre for Mitochondrial Research, Newcastle University, Newcastle upon Tyne, United Kingdom ^2^Institute of Neuroscience, Newcastle University, Newcastle upon Tyne, United Kingdom

## Author Roles

(1) Research Project: A. Conception, B. Organization, C. Execution; (2) Statistical Analysis: A. Design, B. Execution, C. Review and Critique; (3) Manuscript Preparation: A. Writing of the First Draft, B. Review and Critique.

M.H.M.: 1A, 1C, 3A, 3B

G.S.G.: 1A, 1C, 3B

P.G.: 1C, 3B

D.J.B.: 3B

D.M.T.: 3B

A.M.S.: 1C, 3B

## Financial disclosures

M.H.M. is funded by the Sigrid Jusélius Foundation. G.S.G. is funded by the UK National Institute for Health Research (NIHR). D.J.B. is funded by Parkinson's UK, the Michael J. Fox Foundation, the NIHR, and the Wellcome Trust. D.M.T. is funded by the Wellcome Trust.

## Supporting information

Additional Supporting Information may be found in the online version of this article

Supporting Information Video 1

Supporting Information Video 2
